# Injectable antibacterial Ag-HA/ GelMA hydrogel for bone tissue engineering

**DOI:** 10.3389/fbioe.2023.1219460

**Published:** 2023-06-14

**Authors:** Jiapu Wang, Xuefeng Wang, Ziwei Liang, Weiwei Lan, Yan Wei, Yinchun Hu, Longfei Wang, Qi Lei, Di Huang

**Affiliations:** ^1^ Department of Biomedical Engineering, Research Center for Nano-Biomaterials and Regenerative Medicine, College of Biomedical Engineering, Taiyuan University of Technology, Taiyuan, China; ^2^ Shanxi-Zheda Institute of Advanced Materials and Chemical Engineering, Taiyuan, China

**Keywords:** hydroxyapatite microspheres, hydrogel, GelMA, antibacterial, injectable

## Abstract

**Background:** Fracture or bone defect caused by accidental trauma or disease is a growing medical problem that threats to human health.Currently, most orthopedic implant materials must be removed via follow-up surgery, which requires a lengthy recovery period and may result in bacterial infection. Building bone tissue engineering scaffolds with hydrogel as a an efficient therapeutic strategy has outstanding bionic efficiency.By combining some bionic inorganic particles and hydrogels to imitate the organic-inorganic characteristics of natural bone extracellular matrix, developing injectable multifunctional hydrogels with bone tissue repair effects and also displaying excellent antibacterial activity possesses attractive advantages in the field of minimally invasive therapy in clinical.

**Methods:** In the present work, a multifunctional injectable hydrogel formed by photocrosslinking was developed by introducing hydroxyapatite (HA) microspheres to Gelatin Methacryloyl (GelMA) hydrogel.

**Results:** The composite hydrogels exhibited good adhesion and bending resistance properties due to the existence of HA. In addition, when the concentration of GelMA is 10% and the concentration of HA microspheres is 3%, HA/GelMA hydrogel system displayed increased microstructure stability, lower swelling rate, increased viscosity, and improved mechanical properties. Furthermore, the Ag-HA/GelMA demonstrated good antibacterial activity against *Staphylococcus aureus* and *Escherichia coli*, which could signifificantly lower the risk of bacterial infection following implantation. According to cell experiment, the Ag-HA/GelMA hydrogel is capable of cytocompatibility and has low toxicity to MC3T3 cell.

**Conclusion:** Therefore, the new photothermal injectable antibacterial hydrogel materials proposed in this study will provide a promising clinical bone repair strategy and is expected to as a minimally invasive treatment biomaterial in bone repair fields.

## Graphical Abstract

**Figure:**
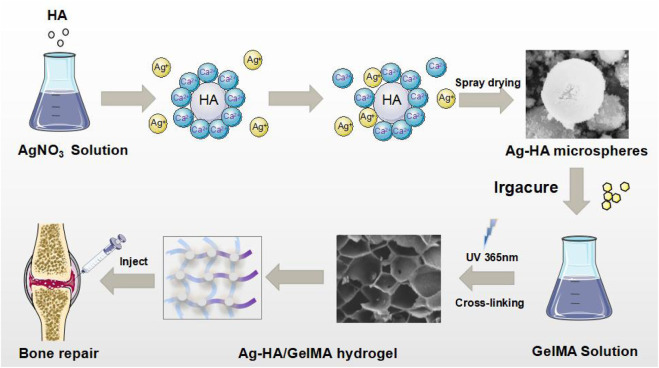
Schematic illustration of the Ag-HA/GelMA hydrogel and the application in bone tissue engineering.

## 1 Introduction

Bone is an important physiological tissue that can regenerate (Salhotra et al., 2020). Because bone’s ability to self-repair is limited, professional intervention is required for fracture or bone defect caused by traumatic injury, bone tumor removal, and bone loss (Dimitriou et al., 2011).With the population aging process accelerated, the question of how to solve the bone repair problem becomes more pressing (Wang et al., 2021; Dong et al., 2022).Clinically, autologous bone transplantation (Schmidt, 2022), allogeneic bone transplantation (Hofmann et al., 1995), and distraction osteogenesis (Papakostidis et al., 2013) are the main treatment for bone regeneration. Although patients readily accept bone materials from themselves, the amount of autologous bone available *in vivo* is limited, and the extraction and treatment process will cause patients additional trauma and pain. At the same time, allogeneic bone transplantation carries risks of infection, bleeding, immune rejection, and disease transmission. Therefore, developing new bone substitutes for bone defect repairing becomes one of the hot issues in the field of minimally invasive therapy.

Artificial bone grafts, such as bioceramics, bioglass, bone cement, hydrogel-based bone tissue engineering scaffolds, and biomaterials are now widely employed in clinical orthopedic surgery (Xue et al., 2022). As a type of polymer material, hydrogel has excellent bionic properties and material adjustability. Hydrogel materials, such as gelatin (Yue et al., 2015), alginate (Saygili et al., 2022), and hyaluronic acid (Park et al., 2020), have emerged as promising candidates for tissue engineering applications.Gelatin can be effectively mixed with natural or synthetic polymers to improve the biological affinity and mechanical properties of scaffold. Methacrylated gelatin, also known as GelMA, is made by adding a methacrylate group to gelatin’s amino group. GelMA has many properties, including biocompatibility, cell adhesion, and adjustable mechanical properties. Because of its similarity to extracellular matrix, it is particularly well suited for the construction of bone tissue engineering scaffolds (Sun et al., 2018; Dong et al., 2019). Nonetheless, the mechanical strength of hydrogels is typically insufficient to meet the load bearing requirements of bone. The mechanical strength of hydrogel can be altered by varying the prepolymer concentration and cross-linking reaction. Cross-linking is a stable process in polymer chemistry that can lead to multidimensional extension of polymer chains, resulting in the formation of network structures. Through cross-linking, the hydrogel forms a stable structure distinct from its constituent ingredients. Hydrogels can be categorized into two groups based on the type of cross-linking bond: chemical cross-link and physical cross-link. Chemically cross-link gels have permanent connections formed by covalent bonds between various polymer chains, resulting in excellent mechanical strength (Shirbhate and Bajaj, 2022). Also, crosslinkers are an important component of the hydrogel system because they can influence pore size, swelling, and elasticity. Therefore, to ensure the materials’ stability under physiological settings, it is crucial to choose the right cross-linking agents based on the type of polymer materials and application.

Some bionic inorganic particles, such as Hydroxyapatite (HA), silicate (Singh et al., 2015) and absorbable bioceramic scaffold (Ma et al., 2018), are widely used in hydrogels to imitate the organic-inorganic characteristics of natural bone extracellular matrix while also improving hydrogel biocompatibility or mechanical properties.Carbon nanotubes, for example, are mixed into hydrogel to make GelMA conductive (Bai et al., 2021). HA is the main inorganic component of human bone tissue (Buckwalter and Cooper, 1987). The addition of HA to the hydrogel can improve the reaction between cells and matrix (Chang et al., 2013; Li et al., 2014), increasing the expression of bone-related genes (Bernhardt et al., 2009). Calcium and phosphorus will be released from the material’s surface and absorbed by body tissues after it is implanted. Aside from encouraging osteoblast adhesion and growth, HA also forms chemical bonds with its own bone, which aids in improving fixation strength with surrounding tissues and allowing for early implant stabilization. In general, the high bio-imitability, strong osteoblast adhesion, excellent mechanical properties and remarkable biocompatibility make HA perfect in bone substitutes applications.

Many different types of hydrogels have been developed as scaffolds for bone tissue engineering, injectable hydrogels have aroused the interest of many researchers. Injectable hydrogels can be molded into a variety of sizes and shapes for bone repair, remodeling, and regeneration (Qiu et al., 2020, Olov et al., 2022), and they can also be used to deliver a variety of ingredients such as stem cells (Muscolino et al., 2021; Bhattacharjee et al., 2022), gene delivery vectors (Ehsanipour et al., 2019; Keeney, 2013), and clinical therapeutic drugs (Yu et al., 2018; Xu et al., 2019). It can be cross-linked *in situ* after injection to form a hydrogel scaffold, which has a good filling effect on complex or fragile bone defects. Thermal gelation, ion interaction, physical self-assembly, photopolymerization, and chemical reaction are commonly used to cross-link injectable hydrogels. Due to the characteristics of photocrosslinking, GelMA is cross-linked and molded by ultraviolet light in the presence of photoinitiator, which can be used as an injectable hydrogel for clinical treatment (Wang et al., 2018; Qiao et al., 2020). The drawback is that its mechanical strength is lower than alginate and polyethylene glycol diacrylate hydrogels and its osteoinductive property is poor. An effective way to solve this problem is to combine organic polymers and inorganic components can give the scaffold greater strength while mimicking the extracellular matrix components of bone cells, as well as promote cell proliferation and differentiation. In this study, a fast-crosslinking injectable hydrogel was developed by combining the unique advantages of GelMA and HA to mimic the natural extracellular matrix of bone. This HA/GelMA hydrogel can not only be injected into the bone defect and cured *in situ* to achieve the filling treatment effect, but it also has a higher compressive capacity and stronger mechanical properties. By incorporating Ag^+^ through ion exchange, the Ag-HA/GelMA exhibits excellent *in vitro* antibacterial antibacterial effects on *S. aureus* (*Staphylococcus aureus*) and *E. coli* (*Escherichia coli*). Furthermore, the Ag-HA/GelMA demonstrated low toxicity and good biocompatibility on MC3T3 cell. In conclusion, this injectable antibacterial Ag-HA/GelMA hydroge scaffold, which mimics the extracellular matrix of bone, provides a good microenvironment for osteoblast culture and is expected to been ploited as a bone tissue engineering material for therapeutic therapy.

## 2 Materials and methods

### 2.1 Materials

Gelatin Porcine Skin, MA and Irgacure 2959 were purchased from Sigma Aldrich. The reagents such as absolute alcohol (purity ≥99.7%), Calcium Nitrate (CaNO_3_) silver nitrate (AgNO_3_) and Trisodium Phosphate (Na_3_PO_4_) were analytical grade and purchased from Tianjin Kaitong Chemical Reagent Co., Ltd., China. The Dulbecco’s modified eagle medium (DMEM) and fetal bovine serum (FBS) were obtained from Sigma Aldrich. Sangon Biotech (Shanghai) Co., Ltd. Supplied trypsin-ethylenediaminetetraacetic acid and phosphate buffer saline (pH 7.4). All of other reagents were analytical grade and used as received without further purification. Mouse embryo osteoblast precursor cells (MC3T3-E1) subclone 14 pre-osteoblasts were sourced from the Shanghai Cell Bank of the Chinese Academy of Sciences.

### 2.2 Synthesis of GelMA

The GelMA were fabricated using a previously reported method (Huang et al., 2021, Miao et al., 2022). First, 20 g gelatin was dissolved in 200 mL PBS at 50°C. After melting the gelatin, slowly add 5 mL MA and let the emulsion rotate at 50°C for 2 h. After stopping the substitution reaction by diluting the reaction mixture with 100 mL PBS, the resulting solution should be dialyzed against deionized water for 7 days using dialysis tubing (8–14 kDa molecular weight cutoff) to ensure complete removal of the low-molecular-weight impurities (including unreacted MA and methacrylic acid byproducts, etc.). Allow dialysis to run for at least 5 days at 40°C using a magnetic stirrer and aluminum foil. Change water 2 times 1 day. Finally, The dialyzed solution can then be freeze dried and kept in a refrigerator or other cold storage until needed.

### 2.3 Synthesis of HA microspheres

HA solutions were prepared from CaNO_3_ and Na_3_PO_4_. The Ca and P precursors were dissolved separately at a ratio of Ca/*p* = 1.67 in equivalent distilled water. Adjust the pH of the Ca solution to 8–10, fill the separatory funnel with the P solution, add dropwise to the Ca solution at a constant speed, and use a booster mechanical stirring pump for strong stirring. The titration time is set to 2 h. During the reaction, keep the pH between 8 and 10. After stopping the stirring, seal the mouth of the beaker with plastic wrap and age for 24 h. Using a spray drying mechanism, HA microspheres were obtained after washing the HA solution with water and centrifugation to remove impurity ions.

### 2.4 Preparation of HA/GelMA hydrogel

The HA microspheres were mixed into GelMA solutions of different concentrations in proportion, and 0.5 w/v% Irgacure 2959 was added for photocrosslinked by UV light. Use 8 × 9 × 3 mm^3^ rectangular PDMS mold to make the mixed solution into a hydrogel. After the solution was injected into the mold, it was irradiated with ultraviolet rays for 60 s to induce gelation. Each hydrogel sample is a rectangular parallelepiped with a height of 3 mm. The sample name and the concentration of each material are shown in Table 1.

**TABLE 1:** The proportion of hybrid hydrogels.

Sample	GelMA (%w/v)	HA microspheres (%w/v)	Irgacure2959 (%w/v)
G5H0	5	0	0.5
G5H1	5	1	0.5
G5H3	5	3	0.5
G5H5	5	5	0.5
G10H0	10	0	0.5
G10H1	10	1	0.5
G10H3	10	3	0.5
G10H5	10	5	0.5
G15H0	15	0	0.5
G15H1	15	1	0.5
G15H3	15	3	0.5
G15H5	15	5	0.5

### 2.5 Preparation of Ag-HA/GelMA hydrogel

The ion exchange method is a process that makes use of the varied ion exchange abilities of exchangeable groups in ion exchange agents and various ions in solution (Dawoud and Abdou, 2022; Torkian et al., 2022). To make the silver-doped HA microspheres antibacterial, the ion exchange method was used for producing silver-containing HA. The hydroxyapatite produced by the reaction of calcium nitrate with trisodium phosphate was immersed in various concentrations of silver nitrate solution and treated by ultrasonic for 5 h, and then the microspheres were obtained by spray drying. The procedure for making hydrogel is the same as in 2.4.

### 2.6 Characterization of the GelMA/HA hydrogel

Phase and microstructure characterization: Scanning electron microscopy (SEM, JEOL JSM7100F, Japan) was used to examine the surface morphology and microstructure of the HA microspheres and GelMA/HA hydrogels after they were sputtered with platinum (Pt). The crystalline phases of the HA microspheres and composite hydrogels were detected by X-ray diffractometer (XRD, Haoyuan 2700BH, China) with a scan speed of 5°/min and a scan step of 0.02°, and scanning range from 10° to 70°. The chemical composition of hybrid hydrogels was identified by Fourier transform infrared spectroscopy (FTIR, Bruker Alpha, Germany) in the range of 4,000–400 cm^−1^.

Swelling tests: To investigate the swelling rate of hybrid hydrogels, the hybrid hydrogels were first freeze-dried and the weight was recorded (W_0_). The lyophilized samples were then immersed in physiological saline (0.9% NaCl) at 37°C. After 24 h, the hydrogels were removed from the physiological saline, the water from the sample surface was gently wiped off with filter paper, and the weight was recorded (W_1_). The swelling ratio of each hydrogel was calculated according to the following formula:

$$Swelling\;ratio\left(100\;\%\right)=\frac{W_{1}-W_{0}}{W_{0}}\times 100\;\%$$

Degradability tests: After recording the initial weight of each sample (Wa), each group of samples was immersed in PBS solution and incubated for 7 days in a shaker (70 rpm) at 37°C. At pre-determined time intervals, samples were removed from PBS and weighed (Wb) after gently removing surface water with filter paper. The formula for calculating the remaining sample weight percent is defined as:

$$Remaining\;mass\left(100\;\%\right)=\frac{W_{b}}{W_{a}}\times 100\;\%$$

Viscosity and fluidity test: The uncured hydrogel samples were stored at 37°C in the sol state. After thoroughly mixing the two phases, the rotary viscometer probe was slowly immersed in the sol to measure the viscosity of each group of materials while waiting for the readings to stabilize. Then, insert the sample into the syringe to see if it can be smoothly extruded.

Mechanical tests: The mechanical properties of hydrogel blocks were investigated using a universal tensile testing machine equipped with a 50 N load sensor (Reger RGM 6030, China). The specimens were prepared in rectangular shape with a dimension of 8 × 9 × 3 mm^3^. In brief, samples were compressed at room temperature at a rate of 1 mm/min until the compression reached 60%, after which the compressive stress-strain curve was obtained. Based on the curve, the compressive strength and compressive modulus (determined as the slope of the linear region corresponding to 5%–10% strain) were calculated. There are five parallel samples in each group.

### 2.7 Antibacterial property of Ag-HA/GelMA hydrogel

Gram-positive *S. aureus* and Gram-negative *E. coli* were chosen as indicators to investigate the antibacterial activity of Ag-HA/GelMA hydrogel. Before the experiments, all disks and materials were sterilized in an autoclave. GelMA, HA/GelMA and Ag-HA/GelMA were coated with various bacterial solutions and placed on agar plates. The samples’ sustained antibacterial activity was assessed by comparing the diameter of an inhibition ring incubated at 37°C for 24 h.

### 2.8 Cytocompatibility of Ag-HA/GelMA hydrogel

The biological properties of GelMA, HA/GelMA and Ag-HA/GelMA hydrogels were analyzed using preosteoblast cell line (MC3T3-E1). Cell proliferation on various hydrogels was evaluated using the Cell Counting Kit8 (CCK-8) assay and live/dead staining. Following sterilization with 75% alcohol and UV light, 1 mL (5×10^3^ cells/mL) MC3T3-E1 cells were seeded on each sample surface in a 24-well plate, and the medium containing cell-hydrogel constructs was incubated at 37°C in a cell incubator filled with 5% CO_2_ for 1, 3, and 5 days, respectively. The original culture medium was removed from the 24-well plate at various time points, then 100 μL of the CCK-8 solution and 900 μL of fresh culture medium were added to each well and incubated at 37°C for 3 h. Finally, 200 μL of the medium was transferred to a 96-well plate, the optical density (OD) values of the resultant solution was measured at 450 nm with a microplate reader (Biorad iMark, United States) to evaluate cell proliferation. The same culture method was used for live/dead staining, with live cells stained green and dead cells stained red. After 30 min in the dark, the hydrogel blocks were washed twice with PBS and examined with a fluorescence inverted microscope (Nikon, TiS, Japan).

### 2.9 Statistical analysis

All experimental data were expressed as mean ± standard deviation (SD), and the statistical analysis was performed using one-way analysis of variance (ANOVA). And *p* < 0.05 was considered statistically significant.

## 3 Results and discussion

### 3.1 Preparation of HA/GelMA hydrogel

**FIGURE 1:**
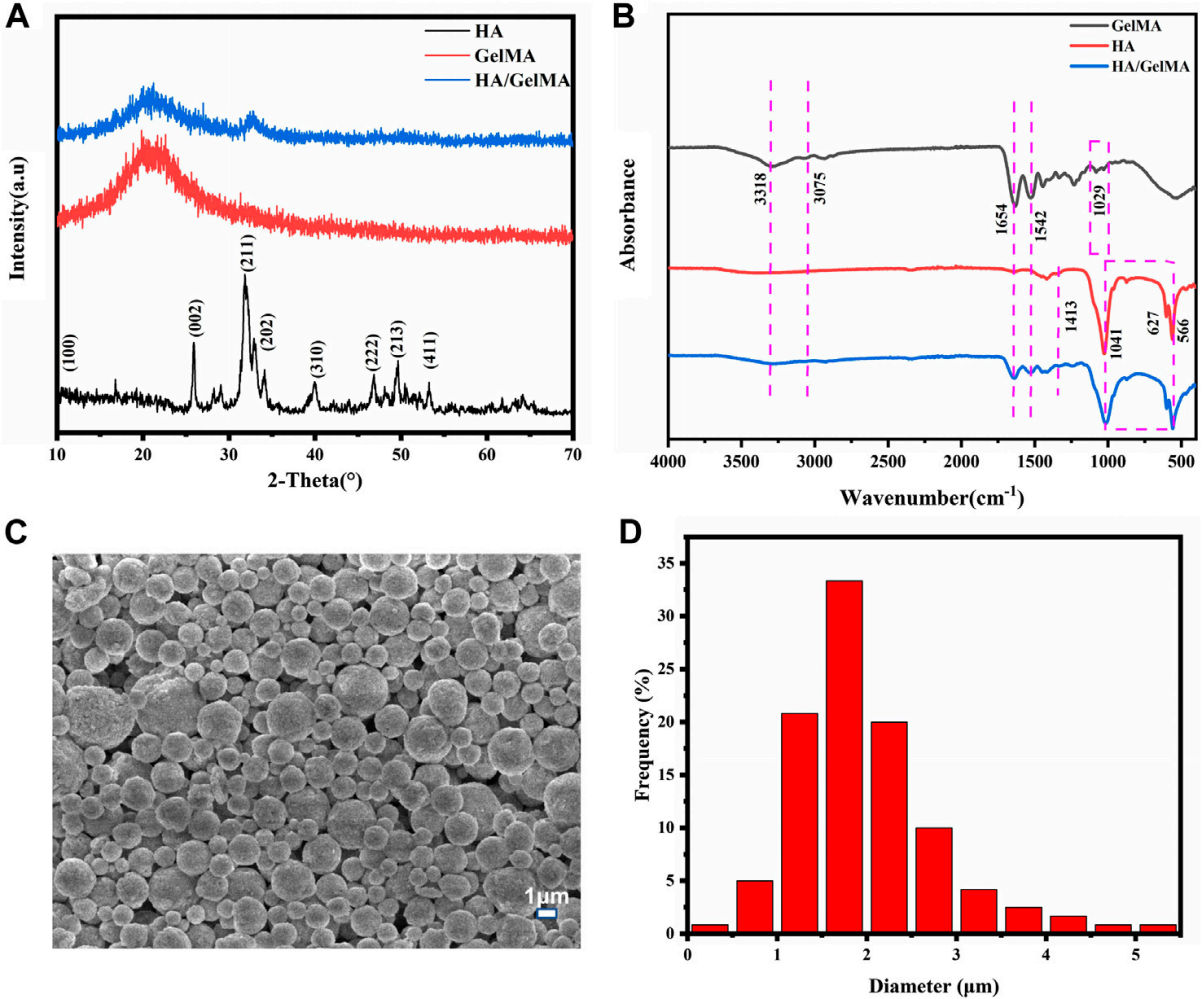
**(A)** The XRD of HA/GelMA hydrogel; **(B)** FTIR spectra of GelMA and HA/GelMA hydrogel; **(C, D)** Microstructure and particle size distribution of HA microspheres.

Presently, the extracellular matrix environment of bone’s complex extracellular matrix cannot be adequately simulated by routinely used orthopedic implants, making them unsuitable for bone regeneration and cell proliferation. Yet, the hydrogel-based bone tissue engineering scaffold can significantly enhance the implant’s characteristics. The organic components of natural bone comprise a significant number of bone glue fibers and a tiny amount of amorphous matrix, and hydrogels have certain similarities with them. Additionally, an organic-inorganic composite has advantages over its individual components in that it can release medication, adapt to the environment, and support the local area until bone regeneration, making them potential substitute for natural bone and provides suitable microenvironment for cells in bone (Soundrapandian C et al., 2009). As a result, the HA/GelMA hydrogel was designed to match the organic-inorganic environmental features of real bone extracellular matrix. We first chemically modified gelatin with methacrylic anhydride and then added an appropriate amount of photoinitiator I2959 to prepare GelMA under ultraviolet light. Then, in order to enhance the scaffold’s mechanical characteristics and osteogenic potential, HA/GelMA hydrogels were created by mixing HA microspheres. Figure 1A depicts the sample XRD spectrum. A new peak was generated at 2θ = 31°–33°, which coincided with the diffraction peak of the (211) and (202) crystal planes of the standard HA spectrum, indicating that HA was successfully compounded with GelMA (Li et al., 2020). According to the results of fourier transform infrared spectroscopy, the A and B bands of amide were located at 3,318 cm^−1^ and 3,075 cm^−1^, respectively, after gelatin was methylated to generate GelMA (Figure 1B).The peak at 1,654 cm^−1^ is mainly caused by the vibration of C=O in the conjugated amide group; and the peak at 1,542 cm^−1^ corresponds to the coupling of N-H bending vibration and C-N stretching vibration. These peaks represent the formation of amide bonds.The C-O-C stretching peak of methacrylic acid group emerges at 1,029 cm^−1^.After GelMA was combined with photoinitiator I2959 and exposed to UV light, the absorption peaks at 3,318 cm^-1^, 3,075 cm^−1^, 1,542 cm^−1^, and 1,029 cm^−1^ were diminished. These results demonstrated that the photocrosslinking reaction of GelMA was successful. Furthermore, the HA/GelMA sample curve shows that characteristic peaks of HA and GelMA coexist and no new peaks are generated, indicating that the two materials are successfully integrated. Many properties of HA are affected by the morphology of the HA crystal, including biological activity, biocompatibility, solubility, sintering, castability, and fracture toughness (Molino et al., 2020; Szcześ et al., 2017).The spray dried HA microspheres in this study have a uniform structure size, a large specific surface area, no agglomeration, and good dispersion and fluidity (Figure 1C). The size of microspheres is concentrated in 1–3 μm, according to the particle size distribution figure obtained from ImageJ statistics (Figure 1D). When the calcium phosphorus ratio of hydrogel material reaches 1.29–1.77, it is considered to be similar to natural bone (Vlad et al., 2012; Boushell et al., 2017; Pal et al., 2023). The EDS analysis revealed a calcium to phosphorus ratio of 1.62, which is quite similar to the natural bone ratio of 1.67 (Supplementary Figure S1). It demonstrates that the HA microspheres can be used for bone repair, providing an inorganic phase for the composite gel.

### 3.2 Morphology of HA/GelMA hydrogel

**FIGURE 2:**
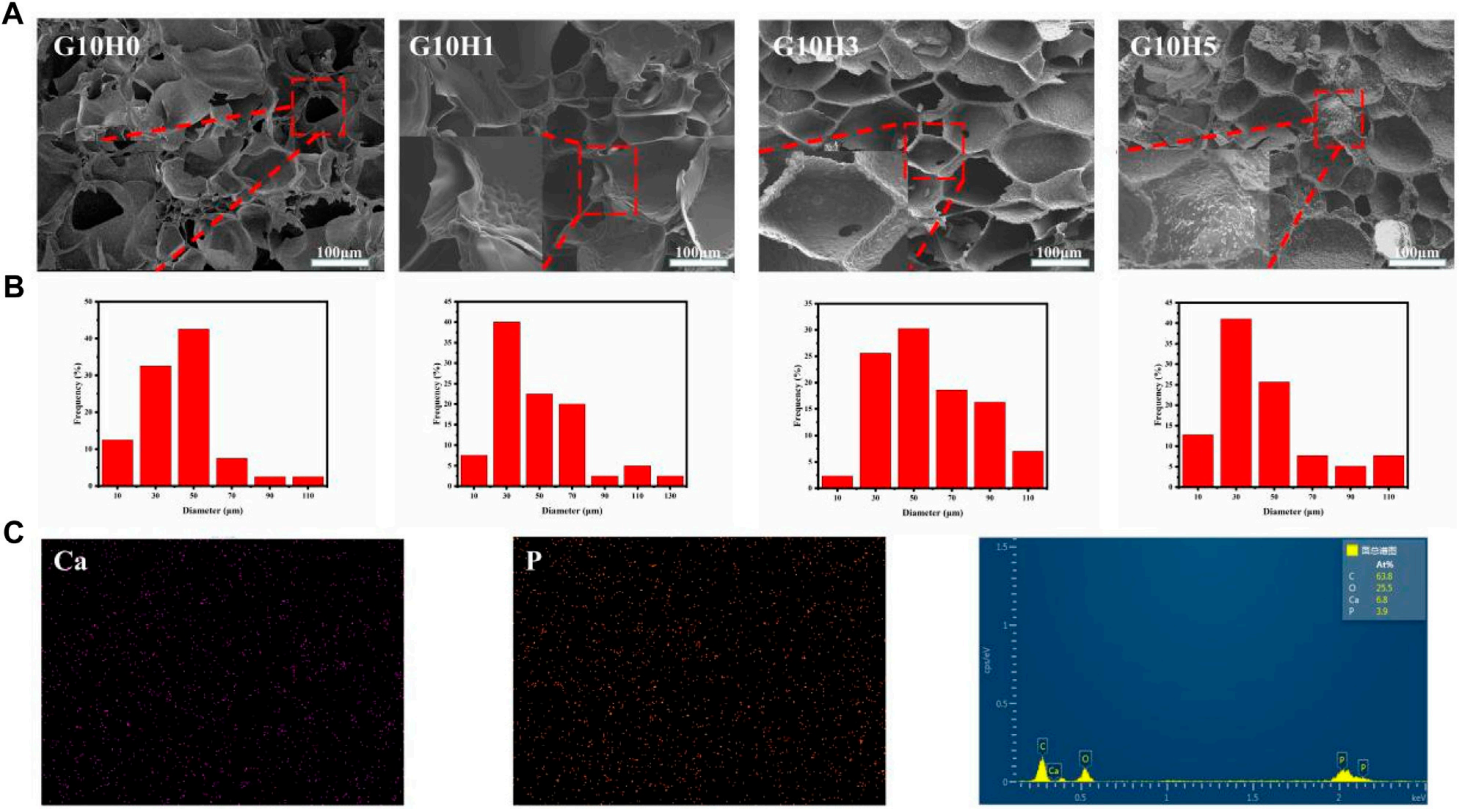
**(A)** SEM images of HA/GelMA hydrogel; **(B)** Pore size distribution histogram of hydrogels in SEM images; **(C)** Element distribution diagram and EDS energy spectrum.

The microstructure of hydrogel can affect its overall efficacy as bone repair material. Hydrogel had a well-defined internal structure that included porous structure and highly interconnected porosity, which can create an ideal microenvironment for cell proliferation (Janoušková et al., 2019; Iglesias-Mejuto and García-González, 2021). The results of SEM showed that the cross section of the composite gel was porous network structure (Supplementary Figure S2), which was conducive to the diffusion of nutrients and the promotion of cell proliferation, and also helped to improve the strength of the hydrogel. When the concentration of GelMA is less than 10%, the pore structure of GelMA alters as HA microsphere concentration rises, indicating that HA concentration can affect pore structure to some extent. When more 5% HA microspheres are added, the pore size changes little, but there are more pores without connectivity, which is inconvenient for material transportation. When the three samples of G5H0, G10H0, and G15H0 are compared, it is clear that increasing the concentration of GelMA results in closer cross-linking. The pore size in the section structure decreases while the pore density increases, resulting in decreased support flexibility and water absorption. Furthermore, when 15% GelMA was mixed with 3% or 5% HA microspheres, the pore size increased significantly, the wall thickness was too thick, and the porosity was reduced. It is possible that the high concentration of GelMA was too thick, and the mixed HA microspheres were not distributed uniformly, preventing the formation of a uniform porous structure (Supplementary Figure S2). As a consequence, the cross section of HA/GelMA hydrogel at 10% mass volume concentration is smoother and better suited for use as a bone repair substance.In the cross sections of G10H1, G10H3 and G10H5, it can be clearly seen that the pore wall of the composite gel is thicker and rougher than that of pure GelMA. HA microspheres are evenly distributed on the pore wall, and cross-linked with GelMA to form an organic-inorganic composite extracellular matrix like structure (Figure 2A). The pore size distribution diagram shows that the pore size of the sample is mostly 10–50 μm without adding HA microspheres, but with the addition of HA microspheres, the pore size increases to 30–70 μm, and has more 90–110 μm macropores (Figure 2B), which is conducive to the transportation of nutrients and waste removal. However, when 5% HA microspheres are added, the pore size decreases and the pore structure becomes irregular. When HA microspheres are present in 3% concentration and GelMA is 10% concentration, the cross section of HA/GelMA hydrogel is smoother and more suitable for use as bone repair materials.Subsequently, EDS analysis was performed on the G10H3 section to evaluate the element composition (Figure 2C). The results revealed that the calcium and phosphorus elements on the surface of the section were evenly distributed. The calcium and phosphorus ratio calculated using the atomic percentage was 6.8:3.9 (1.74), which was close to the calcium and phosphorus ratio of natural HA (1.67). Above all, we conclude that the HA microspheres has a significant impact on the three-dimensional architecture of the hydrogels, particularly the pore size.

### 3.3 Swelling and degradability, viscosity and injectability of HA/GelMA hydrogel

**FIGURE 3:**
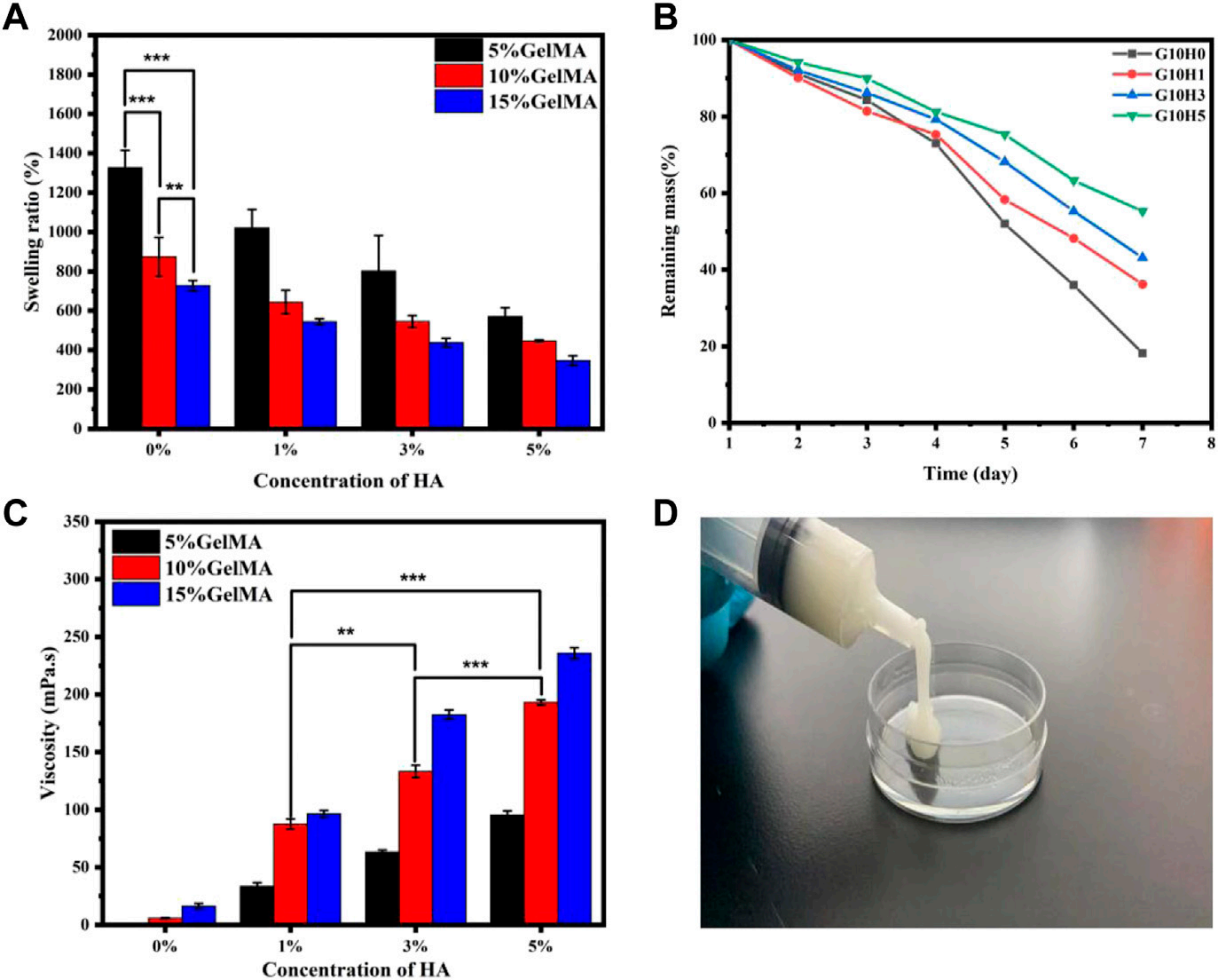
**(A)** The swelling performance of HA/GelMA hydrogel; **(B)** The degradation test of GelMA and HA/GelMA hydrogel; **(C)** The viscosity test of HA/GelMA hydrogel; **(D)** The injectability test of HA/GelMA hydrogel.

The swelling behavior of hydrogels has a direct impact on their hardness, porosity, and hydrophilicity (Park et al., 2006; Zhan et al., 2021). Figure 3A shows that the swelling performance of the HA/GelMA hydrogel gradually decreases as the proportion of HA microspheres increases. G5H0 is the sample with the highest swelling rate, which can reach 1,300%. The swelling rate of G5H5 drops to 600% with a reduction of more than 50% with the addition of 5% HA microspheres. Simultaneously, when the concentration of HA is the same, the higher the concentration of GelMA, the lower the gel swelling rate. G15H3 swelling rate is roughly half that of G5H3. This is due to the high cross-linking reaction density of GelMA, the stent structure being relatively tight, and the pore expansion being poor, resulting in poor water absorption and swelling capacity.

The correct degradation of bone regeneration scaffold is critical for its temporary support (Wei et al., 2020; Bharadwaz and Jayasuriya, 2020). As a result, the degradation performance of the GelMA/HA hydrogel *in vitro* was assessed by soaking it in PBS solution. Figure 3B shows that when the concentration of GelMA is 10%, the degradation behavior of hydrogel differs significantly from that of pure GelMA gel. On the seventh day, G10H0 gel had almost completely degraded, with a weight residual rate of 18.23%. The mass residual rate of G10H5 was the highest, reaching 55.32%, indicating that the addition of HA microspheres significantly reduced hydrogel scaffold degradation. The erosion of the hydrogel is accelerated by the dissolution of chemical bonds, which in turn causes the network structure to become looser. Because the composite scaffold with a higher HA concentration has a higher HA content, it has a higher residual mass percentage after gel component degradation. Simultaneously, the calcium ion in HA can coordinate with the amide bond in the gel, improving the composite hydrogel’s stability and extending its degradation time. As a result, the degradation rate can be adjusted to meet requirements of bone tissue engineering by varying the proportion of GelMA and HA microspheres in the composite hydrogel.

The viscosity test results of the UV curable composite gel are shown in Figure 3C. G10H3 has a viscosity of 133.26 mPa s. The fluidity of the sol is decreased by the inclusion of HA microspheres, making it 51.9% greater than the 87.7 mPa s of G10H1. If the concentration of HA microspheres remains constant, increasing the concentration of GelMA will result in an increase in composite gel viscosity. G10H3 has about twice the viscosity of G5H3, and G15H3 has 55 mPa s more viscosity than G10H3. This is due to the correlation between polymer solution viscosity and concentration. The entanglement of molecular chains and the overlap of molecular clusters grow in high concentration solutions, and the relationship between the concentration and solution viscosity is linear. In conclusion, the viscosity of HA/GelMA hydrogel can be influenced by both the liquid phase concentration and the solid phase concentration, with the liquid phase concentration having a greater influence.

Figure 3D depicts the injectability test results, and the UV curable HA/GelMA hydrogel can be extruded from the syringe at room temperature. The sample has good viscosity and filling properties. It can be quickly cross-linked at the filling site after being irradiated with ultraviolet light, providing a feasible scheme for filling and treating bone defects in clinical applications.

### 3.4 Mechanical properties of HA/GelMA hydrogel

**FIGURE 4:**
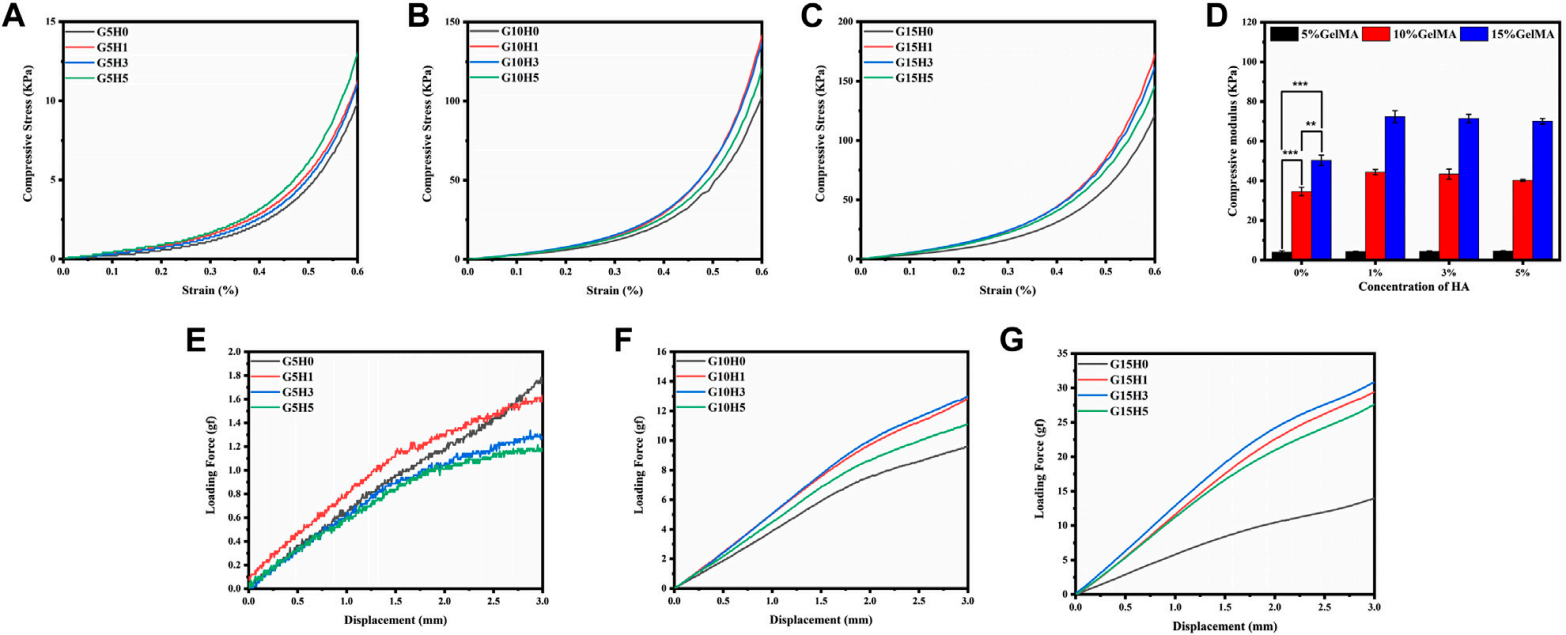
**(A-D)** Compressive mechanical properties of HA/GelMA hydrogel; **(E-G)** Three-point compression test of HA/GelMA hydrogel.

The hydrogel’s primary application possibilities are determined by its mechanical properties (Haq, 2017; Zhu et al., 2019). In comparison to pure gel, the mechanical properties of GelMA hydrogel in combination with inorganic HA microspheres were different. When the concentration of GelMA is 5%, adding HA does not significantly improve the mechanical properties of the composite gel (Figure 4A). When the strain is reduced to 60%, the maximum compressive stress rises from less than 10 kPa–13.81 kPa, while the elastic modulus remains constant at around 4 kPa. This could be because of the low concentration of GelMA, which results in low photocrosslinking strength and the inability to provide a stable support structure.When the GelMA concentration is 10%, the maximum compressive stress of the HA/GelMA hydrogel increases significantly as the HA microsphere content increases (Figure 4B). When the HA microsphere content is 1% and 3%, the maximum compressive stress is 138.24 ± 4.92 kPa. When the HA microsphere content is 5%, the maximum compressive stress drops to 116.23 kPa. This trend is the same when the GelMA concentration is 15% (Figure 4C). The compression modulus of each group of samples was calculated, and the results demonstrate that the concentration of GelMA significantly affects the compression modulus, with an increase in G15H0 relative to G5H0 from 4.23 to 50.14 kPa. When the concentration of GelMA is held constant, the compression modulus of G10H1 increases from 34.62 to 44.43 kPa relative to G10H0, and G15H1 increases by about 20 kPa relative to G15H0 (Figure 4D). The compression modulus decreases slightly as the HA content increases to 3% or 5%, which is the same as the maximum compressive stress test trend. As a result, the mechanical properties of thehydrogel can be altered by varying the HA. At the same time, the mechanical properties of HA/GelMA hydrogel are limited by adding HA microspheres. When high proportion HA is added, the light transmittance decreases and the photocrosslinking strength decreases. Furthermore, because of its crystalline nature, HA can absorb and interfere with ultraviolet radiation, reducing the photocrosslinking effect, and the degree of crosslinking affects the pore size and mechanical strength of HA/GelMA hydrogel.

Implanted bone repair materials must withstand a complex external stress environment that includes different bending and extrusion. The three-point bending test show that the loading force was extremely low and exhibited no bending resistance when GelMA concentration was 5% (Figure 4E). The maximum loading force reaches 13 gf and 31 gf when the concentration of GelMA is 10% and 15% (Figures 4F,G), respectively, and both reach the maximum value of the same group when the concentration of HA microspheres is 1% or 3%, demonstrating that appropriate incorporation of HA microspheres can improve the bending resistance of HA/GelMA hydrogel and make the structure of hydrogel more stable. As a result, this HA/GelMA can accommodate and support bone *in vivo* by improving the mechanical strength and hardness of the hydrogel through HA.

### 3.5 Antibacterial activity of Ag-HA/GelMA

**FIGURE 5:**
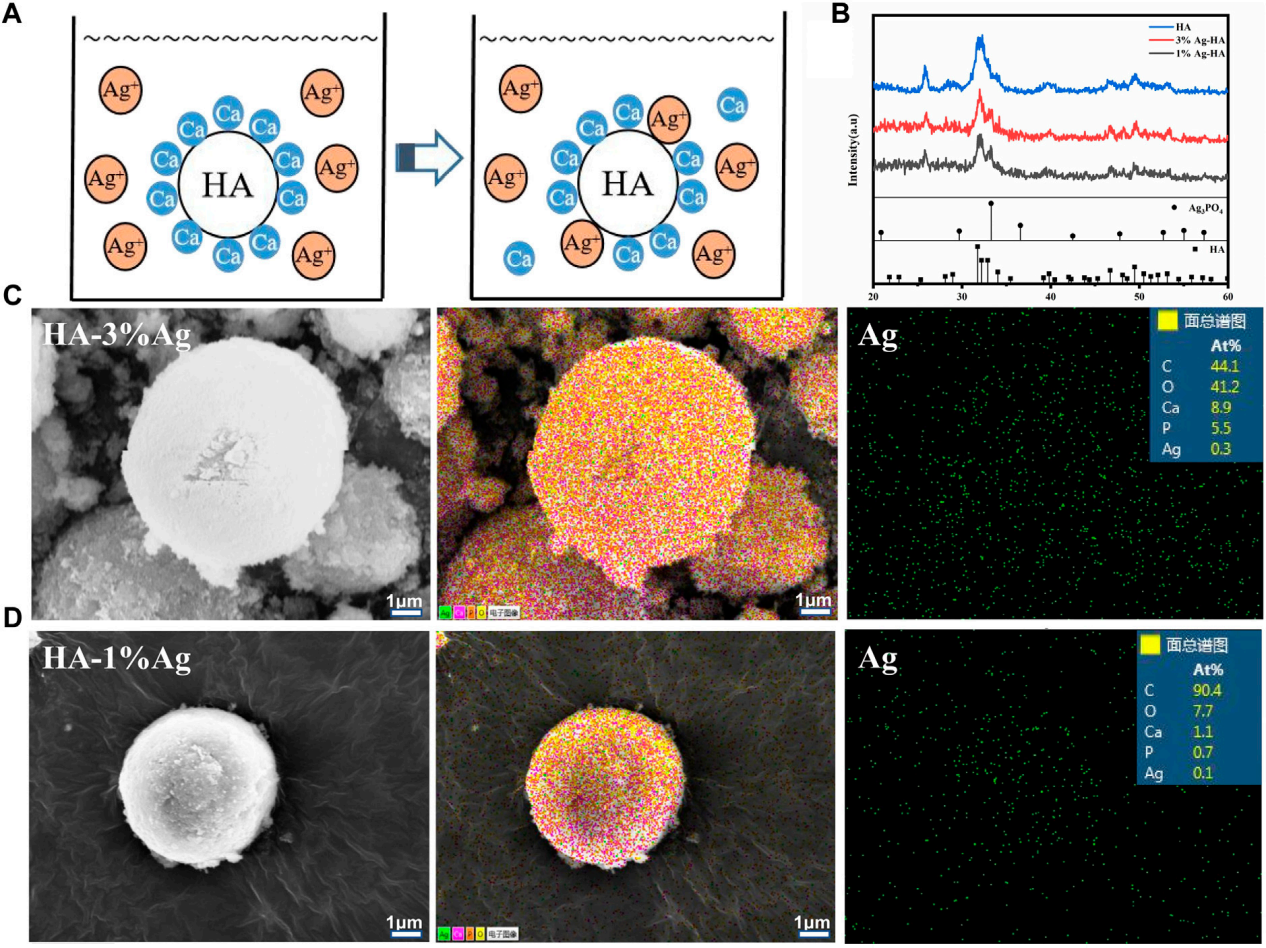
**(A)** Ca element in HA is replaced by Ag element; **(B)** The XRD of silver-doped Ag-HA/GelMA microspheres Microstructure; **(C, D)** Silver element distribution and EDS spectra of silver-doped HA microspheres.

In orthopedic treatment, host immune rejection or bacterial infection caused by implanted materials is a major issue that affects patient health. The antibacterial characteristics of materials can be improved by using antibacterial chemicals or antibacterial particles. To make the silver-doped HA microspheres antibacterial, the ion exchange method was used for producing silver-containing HA. When HA is immersed in silver solution, the Ca element in the lattice can be replaced by Ag element (Figure 5A). Figure 5B depicts the XRD spectrum of silver-doped HA microspheres. The characteristic peaks of silver-doped HA microspheres, such as (002), (211), (202), (310), (222), (213), and (411), are consistent with the standard HA characteristic peaks. When the amount of silver doped is 3%, the phosphate characteristic peaks (200) and (210) were observed at 2θ = 29° and 2θ = 33°, which may be caused by the combination of free phosphate ion and silver ion. When the proportion of silver ion doping is 1%, the characteristic peak of silver phosphate is weak. The SEM images show that the surface of the HA microspheres after silver loading is slightly rough, and the particle size of the HA microspheres has not changed significantly from before (Figures 5C,D). The element distribution diagrams of the two groups of silver-loaded HA microspheres show that calcium, phosphorus, and silver elements are distributed on the surface of the microspheres, with the silver element distribution of the silver-loaded 3% sample being denser than that of the silver-loaded 1% sample.The percentages of silver atoms measured by EDS in the two groups of samples were 0.3% and 0.1%, respectively, which was consistent with the element distribution diagram. The calcium-to-phosphorus ratio on the surface of the two microspheres was 1.62 and 1.57, which was slightly lower than that of natural HA, indicating that some Ca element was successfully replaced by Ag element. Simultaneously, a portion of the Ag^+^ is adsorbed on the surface of the HA microspheres or combined with phosphate to form a trace amount of silver phosphate.

**FIGURE 6:**
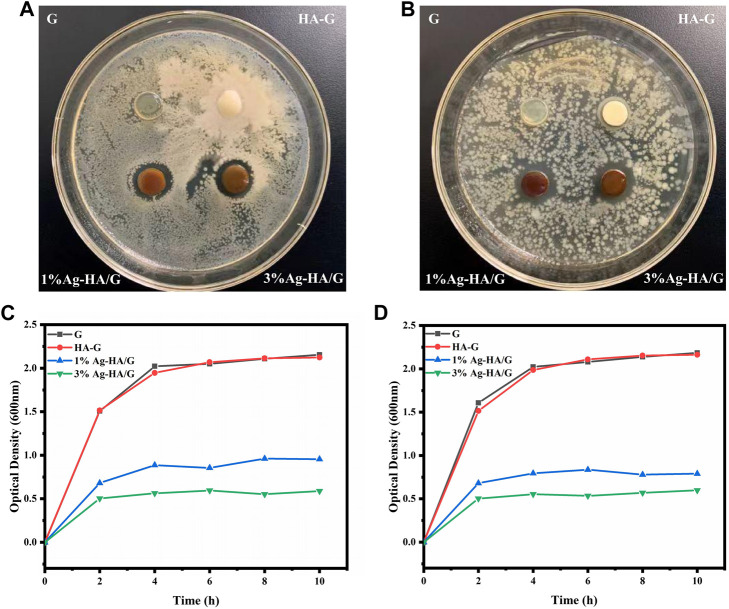
**(A, B)** Experimental results of bacteriostatic zone of Ag-HA/GelMA hydrogel. **(A)** *Staphylococcus aureus*; **(B)** *Escherichia coli*; **(C, D)** Bacteriostatic curve of Ag-HA/GelMA hydrogel. **(C)** *Staphylococcus aureus*; **(D)** *Escherichia coli*.

The antibacterial effect of Ag-HA/GelMA hydrogel on *E. coli* (Gram negative) and *S. aureus* (Gram positive)was then tested using silver-capped HA microspheres combined with GelMA (Figures 6A,B). In both bacteria, two groups of silver-doped samples, 1%Ag-HA/GelMA and 3%Ag-HA/GelMA, showed obvious bacteriostatic circles. Bacteriostatic circles in *S. aureus* were 14.9 and 15.3 mm, while those in *E. coli* were 15.7 and 16.1 mm, respectively. As can be seen, *E. coli* is clearly inhibited by the Ag-HA/GelMA hydrogel, but *S. aureus* is only marginally inhibited, which may be due to the thick cell wall of Gram-positive bacteria and their resistance to antimicrobial agents. The bacteriostatic zone of 1%Ag-HA/GelMA and 3%Ag-HA/GelMA is not significantly different at the same time, indicating that an increase in the amount of silver ions present cannot significantly expand the size of the bacteriostatic zone. Instead, the antibacterial effect is constrained by the interaction between the hydrogel and the bacteria as well as the release of silver ions. Subsequently, we co-cultured *S. aureus* and *E. coli* with four different gels and drew the antibacterial curve of Ag-HA/GelMA hydrogel (Figures 6C,D). Following 2 hours of co-culture, the absorbance of the GelMA and GelMA/HA groups in the two bacterial solutions increased significantly, while 3%Ag-HA/GelMA and 1%Ag-HA/GelMA had good bacteriostatic effect, and 3%Ag-HA/GelMA had better bacteriostatic effect. It suggests that the Ag-HA/GelMA is more suitable for the release of antibacterial HA microspheres during immersion and shaking, where the presence of silver affects the antibacterial capabilities.

### 3.6 Compatibility of Ag-HA/GelMA hydrogel

**FIGURE 7:**
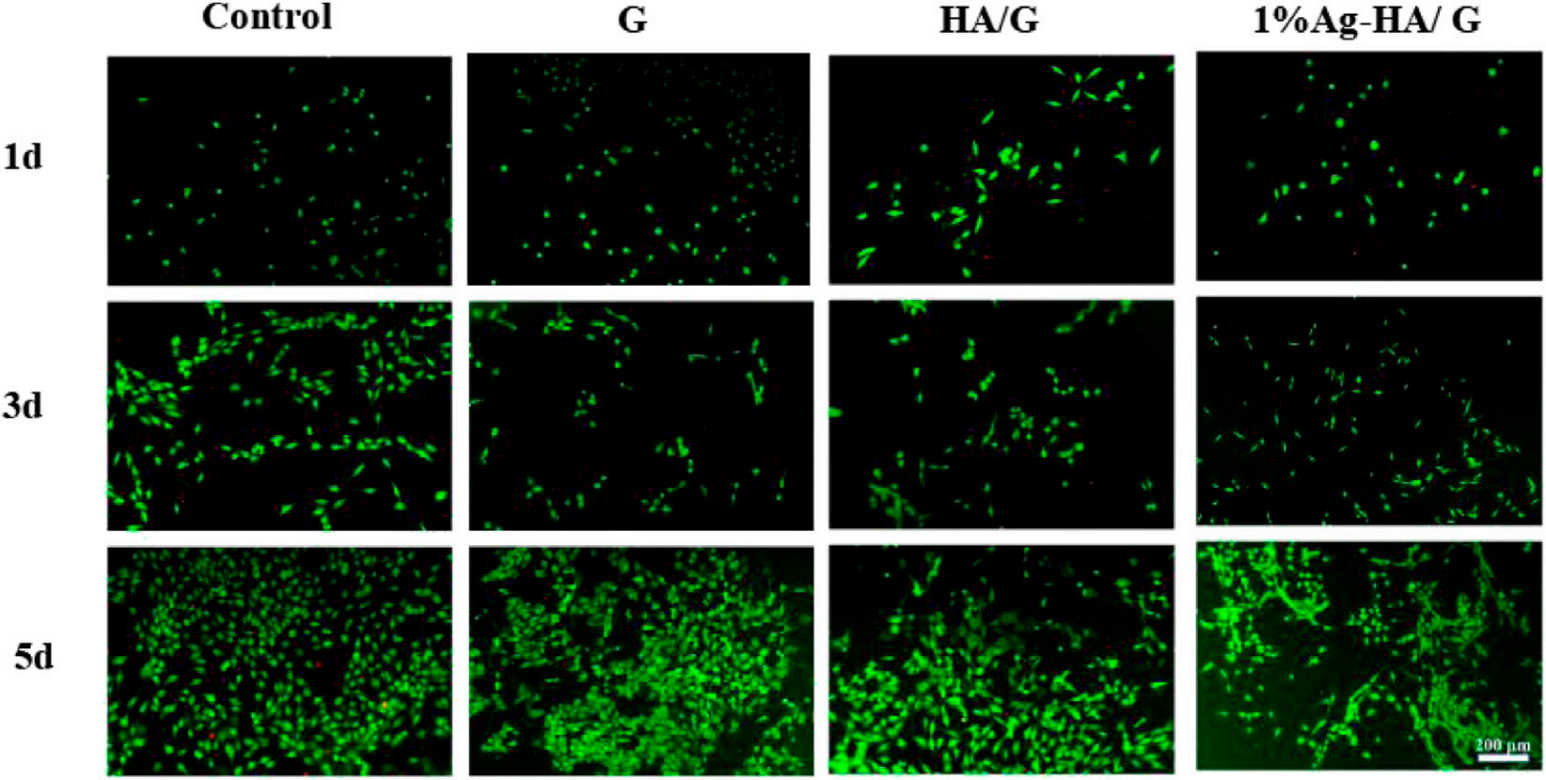
Fluorescence images of Live/Dead staining of MC3T3-E1 cells on the hydrogel surface.

Good biocompatibility is an important characteristic of hydrogels as scaffolds for bone tissue engineering, so it is important to evaluate their toxicity and biocompatibility to mammalian cells. In this test, the cytocompatibility of Ag-HA/GelMA hydrogel was estimated using MC3T3-E1 cells as the normal cells via live and dead staining (Figure 7). The number of cells in the Control, GelMA, HA/GelMA and Ag-HA/GelMA hydrogel increased as culture time increased. The living cells formed pseudopods, indicating that the MC3T3-E1 cells gradually adapted to the hydrogel surface environment. There was no significant difference in the number of living cells observed in four material groups on the first and third days. On the fifth day, many living cells with complete morphology were observed on the surface of HA/GelMA hydrogel, with almost no dead cells; In contrast, the number of living cells on the surface of 1% Ag-HA/GelMA is relatively low, with a few dead cells visible, but the cells continue to proliferate, indicating that trace silver loaded samples have less damage to cells and the materials have no obvious cytotoxicity.

**FIGURE 8:**
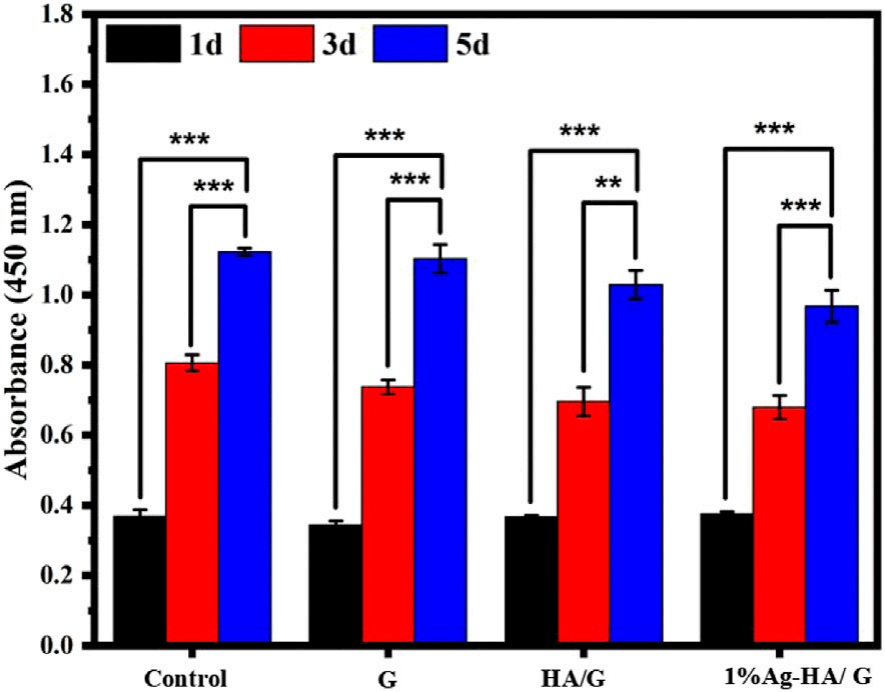
CCK8 assays for proliferation of MC3T3-E1 cells for 1, 3, and 5 days (s).

Furthermore, the CCK-8 experiment confirmed the hydrogel’s cell compatibility (Figure 8). The results showed that the absorbance values of the four groups of samples were not different when the culture time was 1 day and 3 days. As the incubation period approaches 5 days, GelMA hydrogel and Ag-HA/GelMA hydrogel absorbance were slightly lower, but still not lower than 80% of the blank control group. According to the International Organization for Standardization 10,993 standard, when the survival rate of the sample exceeds 70% of the control group, it is considered to be without significant cytotoxicity. Therefore, silver doped HA microspheres bring antibacterial effects while having a slight adverse effect on cells, but the released Ag^+^ concentration is still within the normal range.

## 4 Conclusion

In this work, bionic inorganic particles spray drying HA microspheres were successively introduced into GelMA hydrogel to imitate the organic-inorganic characteristics of natural bone extracellular matrix. The modification of HA exhibited outstanding bending resistance properties and improved mechanical properties, which made the HA/GelMA hydrogel system tough enough to withstand a complex external stress environment. Besides, the hydrogel displays a high injected capacity. After being irradiated with ultraviolet light, it quickly cross-links at the filling site, providing a viable scheme for filling and treating bone defects. Furthermore, the Ag-HA/GelMA exhibits excellent *in vitro* antibacterial antibacterial effects on *S. aureus* and *E. coli* by incorporating Ag element via a “ion exchange” strategy. Furthermore, cell experiments reveal that Ag-HA/GelMA the Ag-HA/GelMA showed low toxicity and good biocompatibility. Therefore, the new photothermal injectable antibacterial hydrogel materials proposed in this study will provide a promising clinical bone repair strategy, and is expected to be applied in the field of minimally invasive bone repair treatment.

## Data Availability

The original contributions presented in the study are included in the article/Supplementary Material, further inquiries can be directed to the corresponding authors.
