# Supplementary material for: Injectable antibacterial Ag-HA/ GelMA hydrogel for bone tissue engineering
Source: Front Bioeng Biotechnol. 2023 Jun 14;11:1219460. doi: 10.3389/fbioe.2023.1219460 (PMC10300446; doi:10.3389/fbioe.2023.1219460)
Supplement: Supplementary file 1 [file DataSheet1.doc]

# Injectable a**ntibacterial** Ag-HA/ GelMA hydrogel for Bone **tissue engineering**

**Jiapu Wang^1,2^,**^†^**, Xuefeng Wang^1^,**^†^**, Ziwei Liang^1,2,*^, Weiwei Lan^1,2^, Yan Wei^1,2^, Yinchun Hu^1,2^, Longfei Wang^1,2^, Qi Lei^1,2^, Di Huang^1,2,*^**

^1^ Department of Biomedical Engineering, Research Center for Nano-Biomaterials & Regenerative Medicine, College of Biomedical Engineering, Taiyuan University of Technology, Taiyuan 030024, PR China;

^2^ Shanxi-Zheda Institute of Advanced Materials and Chemical Engineering, Taiyuan 030032, PR China;

^†^Jiapu Wang^1,2,†^, Xuefeng Wang^1,†^ These authors contributed equally to this work and share first authorship.

**^*^Correspondence:**

**Ziwei Liang**

[liangziweiguozhong@163.com](mailto:liangziweiguozhong@163.com)

**Di Huang**

[huangjw2067@163.com](mailto:huangjw2067@163.com)


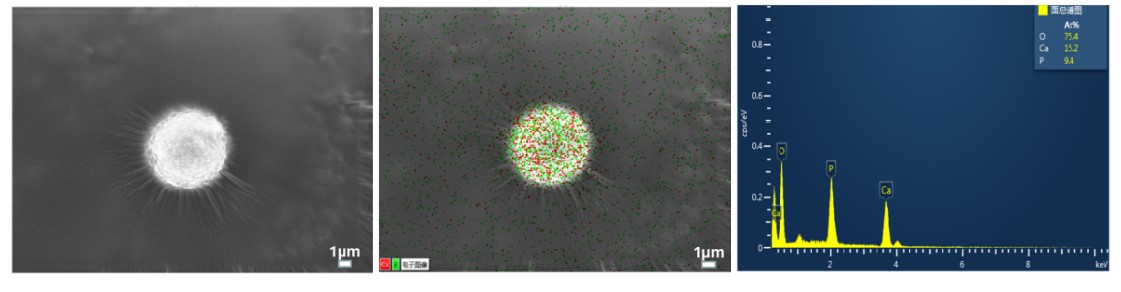


**Supplementary Figure 1.** Surface element distribution and EDS spectra of HA microspheres.


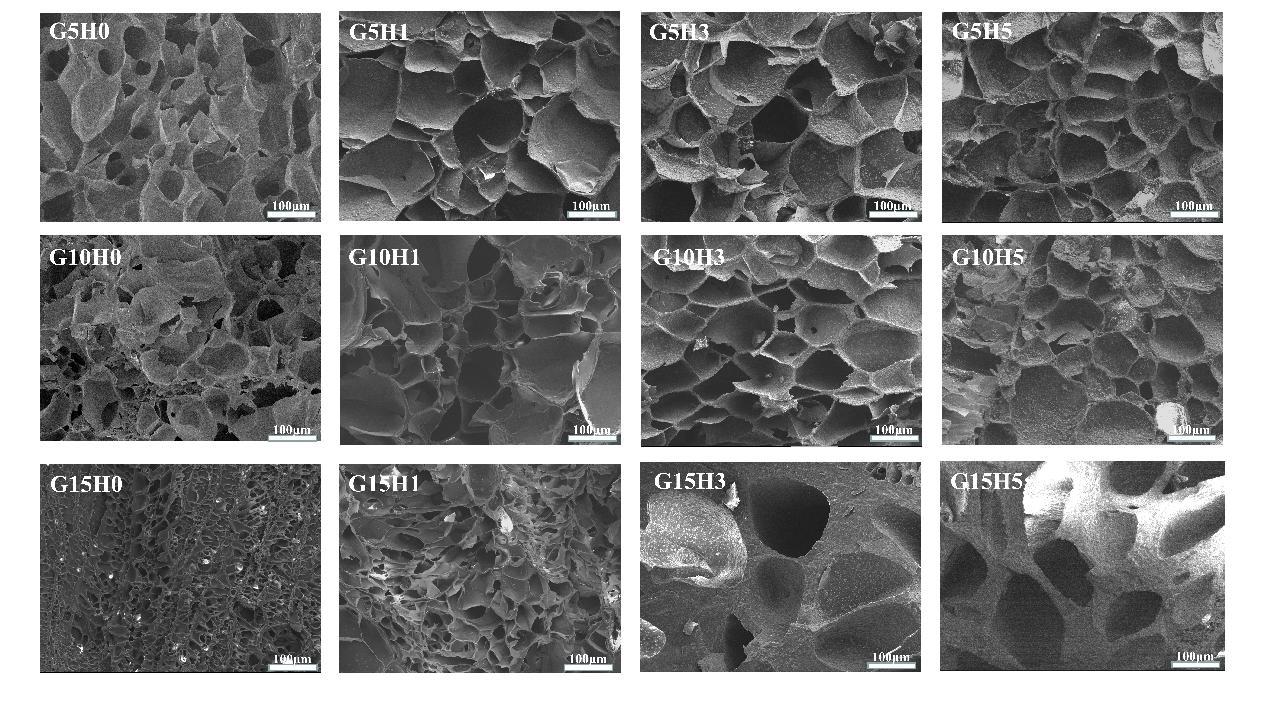


**Supplementary Figure 2.** Microstructure of GelMA/HA composite hydrogel
